# Effects of Oral Vitamin D Supplementation on Physical Performance in Combat-Sport Athletes: A Systematic Review of Randomized Placebo-Controlled Trials

**DOI:** 10.3390/nu18213547

**Published:** 2026-10-28

**Authors:** Marinos Michael, Christoforos D. Giannaki, Pinelopi S. Stavrinou, Eleni Andreou, Angelos Vlahoyiannis

**Affiliations:** 1 Department of Life Sciences, School of Life and Health Sciences, University of Nicosia, Nicosia 2417, Cyprus; marinostcyb@gmail.com (M.M.); giannaki.c@unic.ac.cy (C.D.G.); stavrinou.p@unic.ac.cy (P.S.S.); andreou.el@unic.ac.cy (E.A.); 2 Research Centre for Exercise and Nutrition (RECEN), University of Nicosia, Nicosia 2417, Cyprus

**Keywords:** vitamin D, 25-hydroxyvitamin D, combat sports, physical performance, randomized controlled trial, systematic review, ergogenic aid

## Abstract

Background/Objectives: Vitamin D plays an important role in bone and muscle health; however, whether supplementation improves physical performance in combat-sport athletes remains unclear. This review examined randomized placebo-controlled trials of oral vitamin D supplementation and objective physical-performance outcomes in healthy combat-sport athletes. Methods: PubMed, Scopus, and the Cochrane Library were searched from inception to 22 January 2026, with supplementary targeted verification searches conducted through 1 September 2026. Trials were eligible when combat-sport athletes were randomized to receive vitamin D or placebo/no vitamin D and objective physical performance was assessed. Two reviewers independently performed study selection, data extraction, and risk-of-bias assessment. Findings were synthesized narratively by prioritizing randomized between-group contrasts, group-by-time interactions, numerical changes, and reported effect sizes. Results: Four trials were included, involving 108 randomized participants and 99 analyzed observations. Individual trials reported statistically significant group-by-time effects for selected measures of isokinetic torque, Wingate peak power, high-velocity knee-extension torque, lower-body peak oxygen uptake, handgrip strength, isometric press-up endurance, and lower-body best repeated-sprint peak power. Published standardized effect-size estimates were available for several selected outcomes; however, confidence intervals for between-group effects were generally unavailable. Related outcomes, including Wingate mean or average power, other torque velocities, upper-body peak oxygen uptake, time to exhaustion, oxygen kinetics, wall-sit endurance, and most repeated-sprint measures, showed no statistically significant between-group interaction. No trial assessed competition performance, and all trials presented at least some risk-of-bias concerns. Conclusions: Individual trials reported statistically significant effects on selected laboratory measures. However, the available evidence is not sufficient to determine whether vitamin D supplementation meaningfully improves physical or sport-specific performance in combat-sport athletes. Correcting low vitamin D status should be distinguished from supplementation for performance enhancement. Further well-designed trials are required.

## 1. Introduction

Vitamin D is essential for calcium–phosphate homeostasis, bone mineralization, and musculoskeletal health [1]. Interest in athletic populations also extends to skeletal-muscle and neuromuscular function. Vitamin D-related pathways have been associated with calcium handling, muscle contraction, protein turnover, energy metabolism, inflammation, and muscle remodelling [2,3,4]. These mechanisms are biologically plausible; nevertheless, biological plausibility alone does not demonstrate an ergogenic effect.

Low or suboptimal serum 25-hydroxyvitamin D [25(OH)D] concentrations are frequently reported in athletes, particularly during winter and spring, at higher latitudes, and in athletes who train indoors [5,6,7]. Therefore, the assessment and correction of inadequate vitamin D status may be justified for health. This question should be separated from the stronger claim that vitamin D supplementation improves performance. Performance is determined by multiple factors, including training status, recovery, energy availability, body composition, technical skill, and the specificity and reliability of the test used.

The wider athlete literature remains inconsistent. Vitamin D3 supplementation generally increases serum 25(OH)D when the dose, duration, and adherence are adequate [8,9]. However, the effects on strength and power vary according to baseline vitamin D status, dose, season, participant characteristics, muscle group, and outcome [10,11,12]. A recent meta-analysis of randomized trials found no statistically significant overall effect on maximal strength or power, including in athletes with baseline serum 25(OH)D below 75 nmol/L [9]. Thus, a biochemical response should not be interpreted as evidence of improved physical performance.

Combat sports constitute a relevant but distinct setting. Judo, taekwondo, boxing, wrestling, karate, and mixed martial arts require repeated high-intensity efforts, rapid force production, anaerobic capacity, aerobic recovery, and technical execution under fatigue [13]. Nevertheless, improvements in isokinetic torque, Wingate power, or peak oxygen uptake (VO2peak) do not necessarily translate into better sport-specific or competition performance. The causal contrast is also important. A randomized trial can estimate the effect of vitamin D supplementation only when the randomized groups differ in vitamin D exposure and any training or nutritional cointervention is balanced between groups.

The evidence specific to combat-sport athletes is limited and heterogeneous. A focused synthesis is therefore useful only if direct randomized evidence is separated from uncontrolled pre–post change and if isolated statistically significant outcomes are interpreted in the context of the complete outcome set. The aim of the current systematic review was to evaluate the effects of oral vitamin D supplementation on objective physical-performance outcomes in healthy combat-sport athletes. Outcomes were grouped as neuromuscular or strength-related, anaerobic, and aerobic or endurance-related. Interpretation considered baseline vitamin D status, the supplementation protocol, balanced cointerventions, outcome specificity, risk of bias, and certainty of evidence.

## 2. Materials and Methods

### 2.1. Design and Reporting Framework

The current systematic review of randomized controlled trials was prepared in accordance with the Preferred Reporting Items for Systematic Reviews and Meta-Analyses (PRISMA) 2020 statement [14]. Search reporting was guided by PRISMA-S [15]. Risk of bias was evaluated using the Cochrane Risk of Bias 2 (RoB 2) tool [16], while certainty of evidence was considered using the GRADE domains [17].

### 2.2. Eligibility Criteria

Eligibility was defined using the PICOS framework. Participants were healthy, trained, or competitive combat-sport athletes, including athletes from judo, taekwondo, boxing, wrestling, karate, mixed martial arts, and related disciplines. Studies of non-athletes, clinical or rehabilitation populations, older adults, and military personnel without clearly reported combat-sport participation were excluded.

Eligible interventions were oral vitamin D2 or vitamin D3 at any dose, frequency, or duration. The comparator had to be placebo or no vitamin D. Trials comparing two active vitamin D doses were excluded because they examined the effect of dose rather than supplementation against no supplementation. An additional training or nutritional programme was permitted when it was applied equally to both randomized groups. Trials in which all groups received the same vitamin D regimen were excluded because the comparison between groups did not assess a vitamin D effect.

Eligible outcomes were objective measures of physical performance, including strength or neuromuscular performance, anaerobic performance, aerobic or endurance performance, and sport-related performance tests. Studies reporting only biochemical, immune, microbiome, health, or symptom outcomes were excluded. Only parallel-group randomized placebo-controlled trials were eligible. Fixed-sequence, single-arm, non-randomized, and uncontrolled before–after studies were excluded.

### 2.3. Information Sources and Search Strategy

PubMed, Scopus, and the Cochrane Library were systematically searched from inception to 22 January 2026. Search concepts covered vitamin D, supplementation, athletes, and combat sports. PubMed was limited to human and English-language reports, while Scopus was limited to articles. The formal database search yielded 142 records after filters (PubMed, *n* = 71; Scopus, *n* = 14; Cochrane Library, *n* = 57). Following the formal database search, supplementary targeted searches were conducted during manuscript preparation to identify newly published or closely related reports. These included a broadened PubMed verification search on 1 September 2026, together with examination of reference lists, related reports, ClinicalTrials.gov, and relevant repository records. Four additional reports relevant to the final eligibility criteria were identified through these supplementary methods and assessed using the same eligibility criteria as the formal search. The supplementary search was intended to verify the evidence base and identify relevant newly published reports and did not constitute a complete rerun of Scopus or the Cochrane Library.

### 2.4. Study Selection and Data Collection

Records were exported to EndNote, and automated duplicate identification was followed by manual verification. Two reviewers independently screened titles and abstracts and subsequently assessed potentially eligible reports in full text. Disagreements were resolved through discussion, and reasons for exclusion at the full-text stage were recorded.

Two reviewers independently extracted data using a structured form. Disagreements were resolved through discussion and re-examination of the original report. Extracted data included study design, randomized and analyzed sample sizes, participant and sport characteristics, vitamin D form and dose, intervention duration, comparator and any additional intervention, serum 25(OH)D where reported, performance tests, and numerical results. When several reports described the same study, performance data were extracted from the relevant report and related publications were used to clarify methods or other outcomes. Between-group differences and group-by-time interactions were given priority; changes within one randomized group were not considered evidence of a vitamin D effect. An outcome-by-outcome feasibility assessment found no pair of trials with sufficiently comparable constructs, testing methods, follow-up times, and compatible summary data to support a pooled estimate. Isokinetic torque was assessed at different velocities, Wingate outcomes differed by limb and power metric, aerobic outcomes used different tests or modalities, and repeated-sprint and isometric outcomes were reported by single trials. A meta-analysis was therefore not performed, and the findings synthesized narratively by outcome category.

### 2.5. Risk of Bias and Certainty of Evidence

Two reviewers independently assessed risk of bias using the Cochrane RoB 2 tool [16] for the performance outcomes contributing to each trial’s synthesis. Because RoB 2 is outcome-specific, outcomes were grouped within a trial only when the relevant randomization, deviations from the intended interventions, missing-data, outcome-measurement, and selective-reporting judgments were the same; otherwise, they were assessed separately. Outcome inclusion in the risk-of-bias presentation was not based on statistical significance. Each domain and the overall risk of bias were rated as low risk, some concerns, or high risk. Disagreements were resolved through discussion, and limited reporting was not considered evidence of low risk.

Two reviewers independently assessed the certainty of evidence for each outcome category using the GRADE framework [17] for narrower performance-outcome groups rather than broad composite domains. Disagreements were resolved through discussion. Because pooled estimates were not available, the assessment considered the number and size of trials, consistency of findings across related outcomes, relevance to combat-sport performance, availability of pre-specified analyses, and completeness of the reported results.

## 3. Results

### 3.1. Study Selection

**Figure 1:**
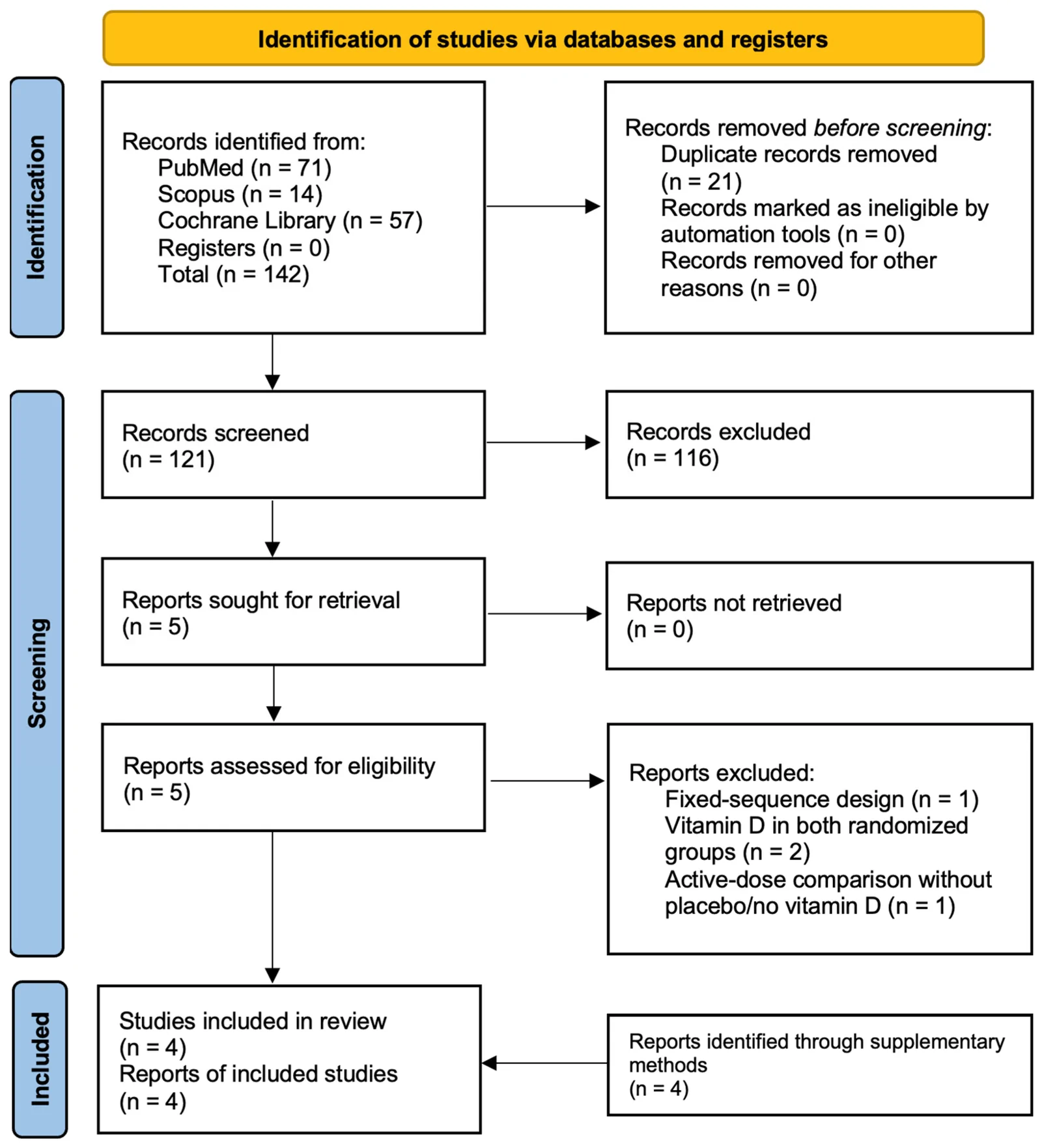
PRISMA 2020 flow diagram of study selection.

Four randomized placebo-controlled trials met the eligibility criteria [18,19,20,21]. The formal database search yielded 142 records; after the removal of 21 duplicates, 121 records were screened, 116 were excluded, and five reports were assessed in full text (Figure 1). One database-derived report was excluded because the population was not eligible. Four additional reports were identified through supplementary targeted methods and assessed in full text; all four were excluded for design or comparator reasons. A related report from the taekwondo trial examined upper-respiratory-tract symptoms and inflammatory markers but did not provide physical-performance data [22]. Four reports from three studies were excluded because they did not compare vitamin D supplementation with a concurrent placebo or no-vitamin-D group [23,24,25,26]. The 2021 Marley study used a fixed placebo-then-supplement sequence rather than randomizing participants to concurrent groups [23]. In the two Przewłócka reports, both randomized groups received vitamin D3 at 3500 IU/day and randomization examined the additional effect of probiotics [24,25]. The Dzik pilot study compared 3500 with 6000 IU/day and did not include a placebo/no-vitamin-D athlete group [26]. Therefore, these studies did not evaluate vitamin D supplementation against placebo or no vitamin D.

### 3.2. Characteristics of Included Studies

The four eligible trials randomized 108 participants, of whom 99 were included in the reported analyses. Participants included national-level male judoka, vitamin D-insufficient collegiate taekwondo athletes, and recreational or sub-elite athletes from mixed martial arts, Brazilian jiu-jitsu, boxing, kickboxing, and karate [18,19,20,21]. Follow-up ranged from 8 days to 6 weeks, and all trials used vitamin D3. Only the trial by Jung et al. included women [19], and none of the trials assessed sport-specific or competition performance. Study characteristics are presented in Table 1.

### 3.3. Risk of Bias

The four included trials were assessed as having some concerns regarding risk of bias [18,19,20,21]. The main concerns were limited reporting of random-sequence generation or allocation concealment, participant dropout in the Jung trial, and the absence of pre-specified protocols or statistical analysis plans for selecting among multiple performance outcomes. For the 2026 Marley trial, some concerns were identified regarding selection of the reported result because no pre-specified protocol or statistical analysis plan was identified and multiple performance outcomes and analyses were reported [21]. The use of objective laboratory tests reduced concerns regarding outcome measurement, but did not eliminate concerns related to selective analysis or reporting. The results of the risk-of-bias assessment are presented in Table 2.

### 3.4. Effects on Vitamin D Status and Physical Performance

The included trials assessed several outcomes related to strength, anaerobic performance, and aerobic or endurance performance. The synthesis below presents the randomized between-group contrast or group-by-time interaction rather than change within one intervention group. Numerical changes and published standardized effect sizes are reported where available. Confidence intervals for the relevant between-group or interaction effects were generally not reported by the trial authors. Because variance of change scores or within-person correlations required for defensible reconstruction were often unavailable, confidence intervals were not generated using unreported assumptions. Statistical significance is therefore presented alongside, rather than as a substitute for, the magnitude and direction of the reported effects (Table 3).

#### 3.4.1. Vitamin D Status

Wyon et al. and Jung et al. reported significant increases in serum 25(OH)D following supplementation [18,19]. In Wyon et al., baseline serum 25(OH)D was 13.2 ± 3.8 ng/mL in the vitamin D group and 16.3 ± 2.7 ng/mL in the placebo group; after 8 days, the corresponding values were 16.8 ± 3.2 and 16.3 ± 2.6 ng/mL [18]. Jung et al. included vitamin D-insufficient athletes who were vitamin D insufficient at baseline; among completers, baseline serum 25(OH)D was 27.3 ± 5.9 nmol/L in the vitamin D group and 30.9 ± 9.4 nmol/L in the placebo group, increasing after intervention to 96.0 ± 3.8 and 32.7 ± 2.4 nmol/L, respectively (group × time *p* < 0.001; partial eta-squared = 0.88) [19]. Neither Marley trial measured serum 25(OH)D [20,21]; therefore, baseline vitamin D status, biochemical response, and adherence to the weekly supplementation protocols were not confirmed biochemically.

#### 3.4.2. Strength and Anaerobic Performance

Wyon et al. reported a treatment-by-time effect for isokinetic torque following a single dose of 150,000 IU dose (*p* = 0.01) [18]. Average muscle strength increased by 13% in the vitamin D group and by approximately 3% in the placebo group. The intervention lasted 8 days, and baseline serum 25(OH)D differed between groups.

Jung et al. reported significant group-by-time interactions for Wingate peak power (F = 7.5, *p* = 0.01, partial eta-squared = 0.19) and knee-extension torque at 180°/s (F = 6.1, *p* = 0.02, partial eta-squared = 0.16) [19]. No statistically significant interactions were reported for Wingate mean power, knee extension and flexion at 60°/s, countermovement jump, sit-ups, agility, or the 20 m pacer test.

During six weeks of identical sprint interval training, Marley et al. reported a group-by-time interaction for lower-body Wingate peak power (*p* = 0.04), with between-group Cohen’s d values for percentage change of 0.52 and 0.62 for sprints 1 and 2, respectively [20]. Upper-body Wingate peak power also showed an interaction (*p* = 0.02), with d = 0.90 and 0.99. By contrast, the group-by-time interactions for lower- and upper-body Wingate average power were not statistically significant (*p* = 0.83 and *p* = 0.42), despite reported between-group d values that varied by sprint. In the 2026 trial, significant interactions were reported for dominant handgrip strength (*p* = 0.04; d_pcc2 = 0.51), and non-dominant handgrip strength (*p* = 0.005; d_pcc2 = 0.62), isometric press-up endurance (*p* = 0.03; d_pcc2 = 0.60), and lower-body best repeated-sprint peak power (*p* = 0.02; d_pcc2 = 0.65) [21]. The wall-sit interaction was not statistically significant (*p* = 0.41; d_pcc2 = 0.28), and most other repeated-sprint outcomes did not show statistically significant group-by-time interactions.

#### 3.4.3. Aerobic and Endurance Performance

Jung et al. reported no statistically significant group-by-time interaction for the 20 m pacer test [19]. In the 2022 Marley trial, lower-body VO2peak increased by 8.2 ± 12% in the vitamin D group and changed by −0.7 ± 11% in the placebo group (interaction *p* = 0.048; d = 0.79) [20]. Upper-body VO2peak did not show a statistically significant interaction (*p* = 0.54; d = 0.48). Lower- and upper-body time to exhaustion improved in both randomized groups, without statistically significant interactions (*p* = 0.43 and *p* = 0.47; d = 0.23 and 0.17, respectively). Oxygen-kinetics outcomes likewise showed no statistically significant between-group interaction (lower body *p* = 0.93, d = 0.12; upper body *p* = 0.37, d = 0.09).

### 3.5. Certainty of Evidence

The certainty of evidence was rated as very low for every outcome group in Table 4. Evidence was downgraded for risk of bias, inconsistency between outcomes, limited relevance to combat-sport performance, and very serious imprecision. Publication bias could not be assessed because only four small trials were included.

## 4. Discussion

The present systematic review is, to our knowledge, the first to examine randomized placebo-controlled trials of oral vitamin D3 supplementation and physical performance specifically in combat-sport athletes. The main findings were that: (i) only four small trials, including 99 analyzed observations, met the eligibility criteria; (ii) significant differences favouring vitamin D were found for selected measures of isokinetic torque, peak power, handgrip strength, press-up endurance, repeated-sprint peak power, and lower-body VO2peak [18,19,20,21], whereas several related outcomes did not differ between groups; (iii) none of the trials assessed sport-specific or competition performance; (iv) baseline vitamin D status and the biochemical response to supplementation were not sufficiently assessed to determine whether they influenced the performance response; and (v) the certainty of evidence was very low for all performance outcomes. Overall, the findings do not support vitamin D3 supplementation as a general strategy to improve performance in combat-sport athletes, although possible effects on selected laboratory measures cannot be excluded.

Previous systematic reviews and meta-analyses have examined vitamin D supplementation in athletes from different sports, with mixed findings for strength and power [9,10,11,12]. A broader systematic review and Bayesian network meta-analysis has also evaluated multiple supplement classes in elite combat-sport athletes [27]. The narrower contribution of the current review is its focus on the causal contrast between oral vitamin D and a concurrent placebo or no-vitamin-D comparator, while excluding fixed-sequence designs, active-dose comparisons, and trials in which both randomized groups received vitamin D. This distinction is important because an improvement in an individual laboratory test may therefore be relevant, but it should not be considered evidence of improved combat-sport performance.

Vitamin D3 supplementation was associated with significant differences in selected measures of isokinetic torque, Wingate peak power, high-velocity knee-extension torque, lower-body VO2peak, handgrip strength, press-up endurance, and lower-body best repeated-sprint peak power [18,19,20,21]. However, several related measures, including mean or average power, other torque velocities, upper-body VO2peak, time to exhaustion, oxygen kinetics, wall-sit endurance, and most repeated-sprint outcomes, did not differ between groups. The available evidence therefore indicates possible effects on specific outcomes rather than a consistent improvement in strength, anaerobic performance, or aerobic capacity.

An important methodological point is how the effect of vitamin D was determined. In both Marley trials, the exercise programme was identical between groups; therefore, the group-by-time interaction represented the additional effect of vitamin D3 alongside training [20,21]. Improvements observed in both groups cannot be attributed to supplementation. In the Przewłócka trial, both randomized groups received the same vitamin D3 dose, and the comparison between groups examined the additional effect of probiotics [24,25]. Similarly, the Dzik trial compared two active vitamin D doses rather than vitamin D with placebo [26]. Using a pre–post change from one group in these studies as evidence of a vitamin D effect would remove the benefit of the randomized comparison and would have the limitations of an uncontrolled before–after analysis.

The large number of outcomes assessed in the included trials also requires careful interpretation. Several studies did not clearly identify a primary outcome or adjust the analyses for multiple comparisons. Therefore, an isolated *p*-value below 0.05 may represent a true effect on a specific outcome, limited statistical power for other outcomes, selective reporting, or chance. The current evidence cannot determine which explanation is most likely. Positive findings should consequently be interpreted with caution, particularly when related performance tests showed no significant differences and effect sizes with confidence intervals were not reported.

The distinction between correcting vitamin D insufficiency and improving performance is also important. Jung et al. included vitamin D-insufficient athletes and reported a clear increase in serum 25(OH)D after supplementation [19]. However, the study did not determine whether athletes with lower baseline vitamin D levels experienced greater improvements in performance. Wyon et al. reported a baseline difference in serum 25(OH)D between groups [18], while neither Marley trial measured serum 25(OH)D [20,21]. As a result, the available evidence cannot identify a baseline vitamin D concentration below which supplementation improves performance or confirm whether the positive findings were limited to athletes with low vitamin D status.

The training programme may also influence the response to supplementation. In the Marley trials, both groups followed the same exercise programme, allowing the additional effect of vitamin D3 to be examined [20,21]. However, the findings remain specific to the participants, supplementation dose, intervention duration, and training programme used, and they should not be generalized to other combat sports, female athletes, longer competitive periods, or competition settings. In addition, the included tests assessed individual components of physical performance rather than combat performance itself. Isokinetic torque, Wingate power, VO2peak, handgrip strength, and repeated-sprint ability are relevant physical characteristics, but they do not include opponent interaction, technical ability, tactical decisions, psychological demands, or weight-category management.

The magnitude and practical importance of the reported effects remain unclear. Most studies focused on statistical significance, while effect sizes and confidence intervals for the differences between groups were rarely reported. A *p*-value does not show whether a difference is large enough or sufficiently precise to influence training or competition. This is particularly important in small studies, where both significant and non-significant findings may be unstable. Future trials should report the difference between groups, the corresponding confidence interval, and, where appropriate, individual responses.

From an applied perspective, assessment and correction of low vitamin D status may be appropriate for health and nutritional adequacy, particularly in athletes with limited ultraviolet-B exposure or other risk factors. This should be distinguished from supplementation specifically intended to improve performance. The included studies used either a single 150,000 IU or 50,000 IU/week, and these protocols should not be directly applied without professional supervision. A weekly dose of 50,000 IU corresponds to approximately 7140 IU/day and exceeds the European Food Safety Authority tolerable upper intake level of 4000 IU/day for habitual adult intake [28]. Supplementation should therefore be based on measured vitamin D status and clinical assessment rather than an expectation of improved performance.

The current review has several strengths. It included only randomized trials that compared vitamin D supplementation with placebo, considered non-significant findings alongside positive results, linked reports from the same study, and distinguished correction of vitamin D status from performance enhancement. However, only four small trials were eligible, and they differed in participant characteristics, supplementation dose, intervention duration, training programme, and performance tests. Formal assessment of small-study effects or funnel-plot asymmetry was not appropriate with so few trials; nevertheless, the evidence is vulnerable to small-study effects. Differences between groups and their confidence intervals were rarely reported. Most participants were men, follow-up was short, and no trial assessed competition performance. It was also not possible to confirm whether participants overlapped between the two Marley trials; therefore, the number of unique participants may be lower than the number of analyzed observations. Lastly, the formal literature search was limited to PubMed, Scopus, and the Cochrane Library, and other potentially relevant bibliographic databases were not searched. In addition, the supplementary search did not constitute a complete synchronized update of the original database search. Therefore, the possibility that an eligible study was not identified cannot be excluded.

Future trials should be prospectively registered, include an adequate sample size, and clearly define the primary outcome. Baseline and post-intervention serum 25(OH)D, adherence, adverse events, and between-group differences with confidence intervals should be reported. Studies are also needed to determine whether baseline vitamin D status influences the response to supplementation. Finally, laboratory tests should be combined with validated combat-sport-specific and, where possible, competition-related outcomes. This would help determine whether the positive findings reported for selected laboratory measures are reproducible and whether they translate into better combat-sport performance.

## 5. Conclusions

Individual trials reported statistically significant effects on selected laboratory outcomes. However, the evidence is insufficient to determine whether vitamin D3 supplementation meaningfully improves physical or sport-specific performance in combat-sport athletes. Findings were not consistent across related outcomes, confidence intervals for randomized-group effects were generally unavailable, and no trial assessed competition performance. Vitamin D supplementation should therefore not be considered a general strategy to improve combat-sport performance. Correcting confirmed low vitamin D status remains important for health and nutritional adequacy, but any additional performance benefit remains uncertain.

## Figures and Tables

**Table 1:** Characteristics of included randomized placebo-controlled trials.

Study	Participant Characteristics and Baseline Vitamin D Status	Sample	Intervention and Comparator	Duration and Training
Wyon et al. [18]	National-level indoor judoka; 22 men. Age: vitamin D group 29 ± 11 y; placebo group 26 ± 7.4 y. Baseline 25(OH)D: 13.2 ± 3.8 vs. 16.3 ± 2.7 ng/mL, respectively.	*n* = 22	Vitamin D3, 150,000 IU once vs. placebo	8 days; no additional training programme reported
Jung et al. [19]	Collegiate taekwondo athletes; 44 randomized (26 men, 18 women), aged 19–22 y; all were vitamin D insufficient (<50 nmol/L) at baseline. Analyzed-sample mean 25(OH)D: 28.8 ± 1.1 nmol/L.	*n* = 44 randomized; 35 completed	Vitamin D3, 5000 IU/day vs. placebo	4 weeks during winter training
Marley et al. [20]	Recreational male combat-sport athletes; 27 men; age 25 ± 5 y. Baseline 25(OH)D not measured.	*n* = 27	Vitamin D3, 50,000 IU/week vs. olive-oil placebo	6 weeks; identical sprint interval training
Marley et al. [21]	Sub-elite male MMA, BJJ, boxing, and karate athletes; 15 men; age 25 ± 4 y. Baseline 25(OH)D not measured.	*n* = 15 (8 vitamin D; 7 placebo)	Vitamin D3, 50,000 IU/week vs. olive-oil placebo	2 weeks; identical hill-sprint and battle-rope training

25(OH)D, 25-hydroxyvitamin D; BJJ, Brazilian jiu-jitsu; IU, international units; MMA, mixed martial arts. Age is reported as mean ± SD unless a range is stated. Sample values are randomized participants; completed/analyzed numbers are shown where attrition occurred.

**Table 2:** Risk-of-bias assessment for selected performance outcomes.

Study and Performance Outcomes Represented	D1	D2	D3	D4	D5	Overall
Wyon et al. [18]: all reported isokinetic quadriceps and hamstring torque outcomes	SC	L	L	L	SC	SC
Jung et al. [19]: Wingate peak power, isokinetic, CMJ, sit-up, agility, and 20 m pacer outcomes	SC	L	SC	L	SC	SC
Marley et al. [20]: VO2peak, TTE, oxygen-kinetics, and Wingate outcomes	SC	L	L	L	SC	SC
Marley et al. [21]: handgrip, isometric-endurance, and repeated-sprint outcomes	SC	L	L	L	SC	SC

D1, randomization process; D2, deviations from intended interventions; D3, missing outcome data; D4, outcome measurement; D5, selection of the reported result; H, high risk; L, low risk; SC, some concerns; RoB 2, Risk of Bias 2.

**Table 3:** Summary of vitamin D status and randomized performance findings.

Study	Vitamin D Status	Reported Numerical Between-Group/Group × Time Findings	Non-Significant Findings and Key Considerations
Wyon et al. [18]	25(OH)D: 13.2 ± 3.8 to 16.8 ± 3.2 ng/mL (vitamin D) vs. 16.3 ± 2.7 to 16.3 ± 2.6 ng/mL (placebo); treatment × time *p* ≤ 0.001.	Isokinetic torque: treatment × time *p* = 0.01; average muscle strength +13% in vitamin D vs. approximately +3% in placebo. Standardized between-group effect and 95% CI not reported.	8-day follow-up; baseline 25(OH)D imbalance; no sport-specific outcome.
Jung et al. [19]	Vitamin D insufficient at baseline; analyzed-sample mean 28.8 ± 1.1 nmol/L. Post-intervention: 96.0 ± 3.8 nmol/L (vitamin D) vs. 32.7 ± 2.4 nmol/L (placebo); group × time *p* < 0.001; partial eta-squared = 0.88.	Wingate peak power: F = 7.5, *p* = 0.01, partial eta-squared = 0.19. Knee extension at 180°/s: F = 6.1, *p* = 0.02, partial eta-squared = 0.16.	No statistically significant group × time interactions for Wingate mean power, other torque velocities, CMJ, sit-ups, agility, or the 20 m pacer test.
Marley et al. [20]	Not measured	Lower-body VO2peak: +8.2 ± 12% vs. −0.7 ± 11%; interaction *p* = 0.048; d = 0.79. Lower-body Wingate peak power: *p* = 0.04; d = 0.52/0.62 (sprints 1/2). Upper-body Wingate peak power: *p* = 0.02; d = 0.90/0.99.	Upper-body VO2peak: *p* = 0.54, d = 0.48. Lower/upper TTE: *p* = 0.43/0.47, d = 0.23/0.17. Lower/upper oxygen kinetics: *p* = 0.93/0.37, d = 0.12/0.09. Wingate average-power interactions were not statistically significant (lower *p* = 0.83; upper *p* = 0.42).
Marley et al. [21]	Not measured	Dominant handgrip: *p* = 0.04, d_pcc2_ = 0.51; non-dominant handgrip: *p* = 0.005, d_pcc2_ = 0.62; isometric press-up: *p* = 0.03, d d_pcc2_ = 0.60; lower-body best repeated-sprint peak power: *p* = 0.02, d_pcc2_ = 0.65.	Wall-sit: *p* = 0.41, d_pcc2 = 0.28. Most other lower- and upper-body repeated-sprint metrics showed no statistically significant group × time interaction; multiple comparisons require cautious interpretation.

25(OH)D, 25-hydroxyvitamin D; CI, confidence interval; CMJ, countermovement jump; d, Cohen’s standardized mean-difference metric for between-group percentage change; TTE, time to exhaustion; VO2peak, peak oxygen uptake. *p*-values are those reported by the trial authors; interaction effect estimates and confidence intervals were generally unavailable.

**Table 4:** Certainty of evidence by physical-performance domain.

Outcome Group	Evidence Base	GRADE Domain Judgments and Reasons	Certainty
Isokinetic muscle torque	Wyon et al. [18]; Jung et al. [19]	Risk of bias: serious (−1), because both contributing trials had some concerns in RoB 2. Inconsistency: serious (−1), because effects differed across velocities and tests (at most *n* = 57 analyzed). Indirectness: not serious (0) for objective physical performance; no inference was made to competition performance. Imprecision: very serious (−2), because the information size was far below that expected for a precise estimate and randomized-group 95% CIs were unavailable. Publication bias: not assessable; no separate downgrade was applied, although small-study and selective-reporting effects remain possible.	Very low
Wingate peak power	Jung et al. [19]; Marley [20]	Risk of bias: serious (−1), because both contributing trials had some concerns in RoB 2. Inconsistency: not serious (0), because both trials reported a group-by-time effect for peak power, although mean/average-power outcomes were less consistent (at most *n* = 62 analyzed). Indirectness: not serious (0) for objective physical performance; no inference was made to competition performance. Imprecision: very serious (−2), because the information size was very small and randomized-group 95% CIs were unavailable. Publication bias: not assessable; no separate downgrade was applied, although small-study and selective-reporting effects remain possible.	Very low
Handgrip and isometric endurance	Marley et al. [21]	Risk of bias: serious (−1), because the trial had some concerns in RoB 2, including the potential influence of multiple outcome testing. Inconsistency: not assessable (one trial). Indirectness: not serious (0) for objective physical performance; no inference was made to competition performance. Imprecision: very serious (−2), because only 15 participants were analyzed and randomized-group 95% CIs were unavailable. Publication bias: not assessable; no separate downgrade was applied, although small-study and selective-reporting effects remain possible.	Very low
Repeated-sprint performance	Marley et al. [21]	Risk of bias: serious (−1), because the trial had some concerns in RoB 2 and reported numerous performance comparisons. Inconsistency: not assessable (one trial). Indirectness: not serious (0) for objective physical performance; no inference was made to competition performance. Imprecision: very serious (−2), because only 15 participants were analyzed and randomized-group 95% CIs were unavailable. Publication bias: not assessable; no separate downgrade was applied, although small-study and selective-reporting effects remain possible.	Very low
VO2peak	Marley et al. [20]	Risk of bias: serious (−1), because the trial had some concerns in RoB 2. Inconsistency: not assessable (one trial). Indirectness: not serious (0) for objective physical performance; no inference was made to competition performance. Imprecision: very serious (−2), because only 27 participants were analyzed and the randomized-group interaction CI was unavailable. Publication bias: not assessable; no separate downgrade was applied, although small-study and selective-reporting effects remain possible.	Very low
Endurance performance (TTE and pacer)	Jung [19]; Marley et al. [20]	Risk of bias: serious (−1), because both contributing trials had some concerns in RoB 2. Inconsistency: not serious (0); neither trial showed a clear randomized-group effect for these endurance measures, although formal heterogeneity could not be estimated (at most *n* = 62 analyzed). Indirectness: not serious (0) for objective physical performance; no inference was made to competition performance. Imprecision: very serious (−2), because the information size was very small and randomized-group 95% CIs were unavailable. Publication bias: not assessable; no separate downgrade was applied, although small-study and selective-reporting effects remain possible.	Very low

## Data Availability

No new data were created or analyzed in this study. Data sharing is not applicable to this article.

## Abbreviations

The following abbreviations are used in this manuscript:

25(OH)D	25-hydroxyvitamin D
BJJ	Brazilian jiu-jitsu
CMJ	Countermovement jump
GRADE	Grading of Recommendations Assessment, Development and Evaluation
IU	International units
MMA	Mixed martial arts
PRISMA	Preferred Reporting Items for Systematic Reviews and Meta-Analyses
RCT	Randomized controlled trial
RoB 2	Risk of Bias 2
SIT	Sprint interval training
VO2peak	Peak oxygen uptake
